# 2-*tert*-Butyl-4-methyl-6-(1,3-oxazinan-1-ylmeth­yl)phenol

**DOI:** 10.1107/S1600536810012109

**Published:** 2010-04-10

**Authors:** Wen-Jun Lei, Shu-Zhong Zhan, Seik Weng Ng

**Affiliations:** a College of Chemistry & Chemical Engineering, South China University of Technology, Guangzhou, 510640, People’s Republic of China; b Department of Chemistry, University of Malaya, 50603 Kuala Lumpur, Malaysia

## Abstract

The title compound, C_16_H_25_NO_2_, which was synthesized by a Mannich reaction route, is a rare example of an organic compound containing the six-membered oxazine ring. The ring adopts a chair conformation and the N atom is pyramidal. The N atom serves as a hydrogen-bond acceptor to the phenolic OH group.

## Related literature

The synthesis from 2-*tert*-butyl-4-methyl­phenol, 3-amino-1-propanol and formaldehyde is an example of carbon–carbon bond formation by the Mannich reaction. For another variation of the Mannich reaction involving 3-amino-1-propanol, see: Korepin *et al.* (2001 ▶).
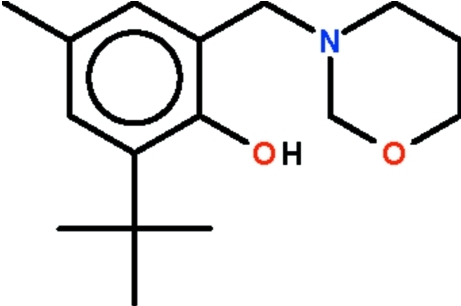


## Experimental

### 

#### Crystal data

C_16_H_25_NO_2_
*M* *_r_* = 263.37
Orthorhombic, 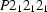

*a* = 6.4740 (7) Å
*b* = 14.1928 (13) Å
*c* = 16.7914 (16) Å
*V* = 1542.9 (3) Å^3^
*Z* = 4
Mo *K*α radiation
μ = 0.07 mm^−1^
*T* = 293 K
0.28 × 0.20 × 0.12 mm

#### Data collection

Rigaku R-AXIS Spider IP diffractometer
Absorption correction: multi-scan (*ABSCOR*; Higashi, 1995 ▶) *T*_min_ = 0.980, *T*_max_ = 0.991
15222 measured reflections
2044 independent reflections
1664 reflections with *I* > 2σ(*I*)
*R*_int_ = 0.022

#### Refinement

*R*[*F* ^2^ > 2σ(*F* ^2^)] = 0.035
*wR*(*F* ^2^) = 0.117
*S* = 1.11
2044 reflections
180 parameters
1 restraint
H atoms treated by a mixture of independent and constrained refinement
Δρ_max_ = 0.14 e Å^−3^
Δρ_min_ = −0.12 e Å^−3^

### 

Data collection: *RAPID-AUTO* (Rigaku, 2002 ▶); cell refinement: *RAPID-AUTO*; data reduction: *CrystalClear* (Rigaku/MSC, 2002 ▶); program(s) used to solve structure: *SHELXS97* (Sheldrick, 2008 ▶); program(s) used to refine structure: *SHELXL97* (Sheldrick, 2008 ▶); molecular graphics: *X-SEED* (Barbour, 2001 ▶); software used to prepare material for publication: *publCIF* (Westrip, 2010 ▶).

## Supplementary Material

Crystal structure: contains datablocks global, I. DOI: 10.1107/S1600536810012109/bt5237sup1.cif

Structure factors: contains datablocks I. DOI: 10.1107/S1600536810012109/bt5237Isup2.hkl

Additional supplementary materials:  crystallographic information; 3D view; checkCIF report

## Figures and Tables

**Table 1:** Hydrogen-bond geometry (Å, °)

*D*—H⋯*A*	*D*—H	H⋯*A*	*D*⋯*A*	*D*—H⋯*A*
O1—H1⋯N1	0.85 (1)	1.90 (2)	2.665 (2)	149 (3)

**Fig. 1.**
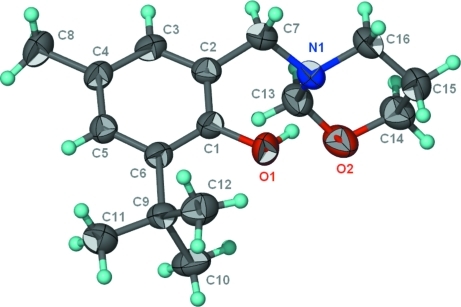
Anisotropic displacement ellipsoid plot (Barbour, 2001) of the title compound at the 50% probability level; hydrogen atoms are drawn as spheres of arbitrary radius.

## supplementary crystallographic information

### Comment

Organic synthesis centers largely on stereoselective carbon–carbon and carbon–heteroatom bond-forming reactions; among such reactions is the class of Mannich reactions, which can be regarded as being the most important carbon–carbon bond-forming reaction. The reactions lead to β-aminocarbonyl compounds, which are important intermediates for pharmaceuticals.

One variation of the Mannich reaction involves the catalytic addition of an amine, R_2_NH, to an alkene or alkyne, i. e., hydroamination. In the 2-*tert*-butyl-4-methylphenol reacts with 3-amino-1-propanol to yield a compound having a 1,3-oxazinyl ring (Scheme I, Fig. 1). Such a ring is difficult to synthesis by conventional routes.

### Experimental

2-*tert*-Butyl-4-methylphenol (2.24 g, 12.3 mmol), 3-amino-1-propanol (0.93 g, 12.3 mmol), 37% aqueous formaldehyde (1.83 ml, 24.6 mmol) and triethylamine (2.49 g, 24.6 mmol) in ethanol (50 ml) were heated for 6 hours. Slow evaporation of the filtrate gave light-yellow crystals in 70% yield.

### Refinement

Carbon-bound H-atoms were allowed to ride on their parent atoms (C–H 0.93– 0.97 Å) and their displacement parameters were set to  1.2–1.5U_eq_(C). The hydroxy H-atom was  located in a difference Fourier map, and was refined isotropically with a distance restraint of O–H 0.84±0.01 Å.

Due to the absence of anomalous scatterers, the absolute configuration could not be determined, and, therefore, 1488 Friedel pairs were merged.

### Crystal data

**Table:**

C_16_H_25_NO_2_	*F*(000) = 576
*M**_r_* = 263.37	*D*_x_ = 1.134 Mg m^−^^3^
Orthorhombic, *P*2_1_2_1_2_1_	Mo *K*α radiation, λ = 0.71073 Å
Hall symbol: P 2ac 2ab	Cell parameters from 12093 reflections
*a* = 6.4740 (7) Å	θ = 3.1–27.5°
*b* = 14.1928 (13) Å	µ = 0.07 mm^−^^1^
*c* = 16.7914 (16) Å	*T* = 293 K
*V* = 1542.9 (3) Å^3^	Block, yellow
*Z* = 4	0.28 × 0.20 × 0.12 mm

### Data collection

**Table:**

Rigaku R-AXIS Spider IP diffractometer	2044 independent reflections
Radiation source: fine-focus sealed tube	1664 reflections with *I* > 2σ(*I*)
graphite	*R*_int_ = 0.022
ω scan	θ_max_ = 27.5°, θ_min_ = 3.1°
Absorption correction: multi-scan (*ABSCOR*; Higashi, 1995)	*h* = −8→8
*T*_min_ = 0.980, *T*_max_ = 0.991	*k* = −18→18
15222 measured reflections	*l* = −21→21

### Refinement

**Table:**

Refinement on *F*^2^	Primary atom site location: structure-invariant direct methods
Least-squares matrix: full	Secondary atom site location: difference Fourier map
*R*[*F*^2^ > 2σ(*F*^2^)] = 0.035	Hydrogen site location: inferred from neighbouring sites
*wR*(*F*^2^) = 0.117	H atoms treated by a mixture of independent and constrained refinement
*S* = 1.11	*w* = 1/[σ^2^(*F*_o_^2^) + (0.0739*P*)^2^ + 0.0371*P*] where *P* = (*F*_o_^2^ + 2*F*_c_^2^)/3
2044 reflections	(Δ/σ)_max_ = 0.001
180 parameters	Δρ_max_ = 0.14 e Å^−^^3^
1 restraint	Δρ_min_ = −0.12 e Å^−^^3^

### Special details

**Table:**

Geometry. All esds (except the esd in the dihedral angle between two l.s. planes) are estimated using the full covariance matrix. The cell esds are taken into account individually in the estimation of esds in distances, angles and torsion angles; correlations between esds in cell parameters are only used when they are defined by crystal symmetry. An approximate (isotropic) treatment of cell esds is used for estimating esds involving l.s. planes.

### Fractional atomic coordinates and isotropic or equivalent isotropic displacement parameters (Å^2^)

**Table:**

	*x*	*y*	*z*	*U*_iso_*/*U*_eq_	
O1	0.8894 (2)	0.48173 (11)	0.62162 (8)	0.0654 (4)	
H1	0.972 (4)	0.5284 (14)	0.6242 (18)	0.095 (9)*	
O2	0.9444 (3)	0.75355 (12)	0.59189 (10)	0.0816 (5)	
N1	1.0668 (3)	0.63695 (11)	0.67909 (9)	0.0550 (4)	
C1	0.7719 (3)	0.47819 (12)	0.68939 (10)	0.0494 (4)	
C2	0.8294 (3)	0.53321 (12)	0.75571 (10)	0.0506 (4)	
C3	0.7081 (3)	0.53034 (12)	0.82338 (10)	0.0536 (4)	
H3	0.7451	0.5670	0.8670	0.064*	
C4	0.5332 (3)	0.47461 (12)	0.82833 (10)	0.0524 (4)	
C5	0.4816 (3)	0.42039 (12)	0.76175 (10)	0.0501 (4)	
H5	0.3656	0.3819	0.7646	0.060*	
C6	0.5953 (3)	0.42121 (12)	0.69126 (10)	0.0468 (4)	
C7	1.0274 (3)	0.58874 (15)	0.75529 (11)	0.0594 (5)	
H7A	1.0222	0.6353	0.7975	0.071*	
H7B	1.1415	0.5465	0.7666	0.071*	
C8	0.4047 (4)	0.47229 (16)	0.90293 (11)	0.0731 (6)	
H8A	0.2749	0.4423	0.8919	0.110*	
H8B	0.3806	0.5355	0.9211	0.110*	
H8C	0.4765	0.4376	0.9434	0.110*	
C9	0.5275 (3)	0.36320 (13)	0.61807 (10)	0.0539 (4)	
C10	0.4755 (5)	0.43096 (16)	0.54936 (12)	0.0754 (6)	
H10A	0.3555	0.4673	0.5630	0.113*	
H10B	0.4484	0.3953	0.5019	0.113*	
H10C	0.5901	0.4725	0.5403	0.113*	
C11	0.3358 (4)	0.30415 (17)	0.63527 (14)	0.0745 (6)	
H11A	0.2244	0.3448	0.6510	0.112*	
H11B	0.3651	0.2605	0.6774	0.112*	
H11C	0.2971	0.2701	0.5882	0.112*	
C12	0.7001 (4)	0.29608 (16)	0.59218 (13)	0.0731 (6)	
H12A	0.6504	0.2558	0.5504	0.110*	
H12B	0.7423	0.2584	0.6368	0.110*	
H12C	0.8157	0.3320	0.5731	0.110*	
C13	0.9229 (3)	0.71409 (15)	0.66757 (13)	0.0668 (5)	
H13A	0.7828	0.6912	0.6742	0.080*	
H13B	0.9476	0.7621	0.7076	0.080*	
C14	1.1466 (4)	0.79381 (18)	0.58285 (17)	0.0839 (7)	
H14A	1.1654	0.8439	0.6215	0.101*	
H14B	1.1600	0.8208	0.5300	0.101*	
C15	1.3100 (4)	0.71942 (18)	0.59488 (14)	0.0743 (6)	
H15A	1.3017	0.6732	0.5524	0.089*	
H15B	1.4457	0.7482	0.5930	0.089*	
C16	1.2798 (3)	0.67164 (15)	0.67383 (13)	0.0633 (5)	
H16A	1.3757	0.6195	0.6790	0.076*	
H16B	1.3065	0.7158	0.7167	0.076*	

### Atomic displacement parameters (Å^2^)

**Table:**

	*U*^11^	*U*^22^	*U*^33^	*U*^12^	*U*^13^	*U*^23^
O1	0.0583 (9)	0.0845 (9)	0.0533 (7)	−0.0116 (8)	0.0156 (7)	−0.0123 (7)
O2	0.0656 (10)	0.0945 (10)	0.0847 (10)	−0.0108 (9)	−0.0202 (8)	0.0295 (9)
N1	0.0417 (8)	0.0652 (8)	0.0579 (8)	−0.0029 (7)	−0.0030 (7)	0.0015 (7)
C1	0.0461 (9)	0.0594 (8)	0.0427 (8)	0.0024 (8)	0.0025 (7)	−0.0022 (7)
C2	0.0480 (10)	0.0563 (9)	0.0474 (8)	0.0045 (8)	−0.0048 (7)	0.0002 (8)
C3	0.0618 (11)	0.0578 (9)	0.0412 (8)	0.0048 (9)	−0.0042 (8)	−0.0049 (7)
C4	0.0547 (10)	0.0583 (8)	0.0443 (8)	0.0077 (8)	0.0048 (8)	−0.0005 (8)
C5	0.0474 (10)	0.0540 (8)	0.0491 (8)	0.0026 (8)	0.0026 (7)	0.0007 (7)
C6	0.0456 (9)	0.0517 (8)	0.0431 (7)	0.0058 (7)	−0.0011 (7)	−0.0018 (7)
C7	0.0526 (11)	0.0719 (10)	0.0537 (9)	−0.0032 (10)	−0.0084 (9)	0.0020 (9)
C8	0.0795 (16)	0.0877 (13)	0.0519 (11)	0.0033 (13)	0.0197 (10)	−0.0034 (10)
C9	0.0553 (11)	0.0637 (10)	0.0428 (8)	0.0006 (9)	−0.0051 (8)	−0.0044 (8)
C10	0.0868 (17)	0.0854 (13)	0.0541 (10)	−0.0005 (13)	−0.0201 (11)	0.0065 (10)
C11	0.0764 (16)	0.0823 (13)	0.0649 (12)	−0.0191 (12)	−0.0065 (11)	−0.0106 (11)
C12	0.0834 (17)	0.0754 (12)	0.0606 (11)	0.0122 (13)	0.0005 (11)	−0.0172 (10)
C13	0.0492 (11)	0.0760 (11)	0.0752 (13)	0.0046 (10)	−0.0018 (11)	0.0095 (11)
C14	0.0771 (17)	0.0884 (14)	0.0861 (16)	−0.0235 (14)	−0.0163 (14)	0.0215 (13)
C15	0.0625 (14)	0.0911 (14)	0.0694 (13)	−0.0203 (12)	0.0040 (11)	−0.0028 (11)
C16	0.0458 (10)	0.0732 (11)	0.0708 (12)	−0.0052 (9)	−0.0033 (10)	−0.0011 (10)

### Geometric parameters (Å, °)

**Table:**

O1—C1	1.370 (2)	C9—C11	1.526 (3)
O1—H1	0.852 (10)	C9—C12	1.531 (3)
O2—C13	1.396 (3)	C9—C10	1.539 (3)
O2—C14	1.436 (3)	C10—H10A	0.9600
N1—C13	1.450 (3)	C10—H10B	0.9600
N1—C16	1.467 (3)	C10—H10C	0.9600
N1—C7	1.473 (2)	C11—H11A	0.9600
C1—C6	1.401 (3)	C11—H11B	0.9600
C1—C2	1.410 (2)	C11—H11C	0.9600
C2—C3	1.382 (3)	C12—H12A	0.9600
C2—C7	1.505 (3)	C12—H12B	0.9600
C3—C4	1.384 (3)	C12—H12C	0.9600
C3—H3	0.9300	C13—H13A	0.9700
C4—C5	1.398 (2)	C13—H13B	0.9700
C4—C8	1.504 (2)	C14—C15	1.508 (4)
C5—C6	1.394 (2)	C14—H14A	0.9700
C5—H5	0.9300	C14—H14B	0.9700
C6—C9	1.543 (2)	C15—C16	1.502 (3)
C7—H7A	0.9700	C15—H15A	0.9700
C7—H7B	0.9700	C15—H15B	0.9700
C8—H8A	0.9600	C16—H16A	0.9700
C8—H8B	0.9600	C16—H16B	0.9700
C8—H8C	0.9600		
			
C1—O1—H1	110 (2)	C9—C10—H10B	109.5
C13—O2—C14	110.29 (18)	H10A—C10—H10B	109.5
C13—N1—C16	110.01 (15)	C9—C10—H10C	109.5
C13—N1—C7	110.80 (16)	H10A—C10—H10C	109.5
C16—N1—C7	111.79 (16)	H10B—C10—H10C	109.5
O1—C1—C6	119.54 (15)	C9—C11—H11A	109.5
O1—C1—C2	119.30 (17)	C9—C11—H11B	109.5
C6—C1—C2	121.15 (16)	H11A—C11—H11B	109.5
C3—C2—C1	118.89 (18)	C9—C11—H11C	109.5
C3—C2—C7	120.23 (16)	H11A—C11—H11C	109.5
C1—C2—C7	120.73 (17)	H11B—C11—H11C	109.5
C2—C3—C4	122.09 (16)	C9—C12—H12A	109.5
C2—C3—H3	119.0	C9—C12—H12B	109.5
C4—C3—H3	119.0	H12A—C12—H12B	109.5
C3—C4—C5	117.54 (16)	C9—C12—H12C	109.5
C3—C4—C8	121.00 (16)	H12A—C12—H12C	109.5
C5—C4—C8	121.45 (18)	H12B—C12—H12C	109.5
C6—C5—C4	123.24 (18)	O2—C13—N1	111.13 (18)
C6—C5—H5	118.4	O2—C13—H13A	109.4
C4—C5—H5	118.4	N1—C13—H13A	109.4
C5—C6—C1	117.06 (15)	O2—C13—H13B	109.4
C5—C6—C9	121.45 (17)	N1—C13—H13B	109.4
C1—C6—C9	121.49 (15)	H13A—C13—H13B	108.0
N1—C7—C2	113.26 (15)	O2—C14—C15	110.27 (18)
N1—C7—H7A	108.9	O2—C14—H14A	109.6
C2—C7—H7A	108.9	C15—C14—H14A	109.6
N1—C7—H7B	108.9	O2—C14—H14B	109.6
C2—C7—H7B	108.9	C15—C14—H14B	109.6
H7A—C7—H7B	107.7	H14A—C14—H14B	108.1
C4—C8—H8A	109.5	C16—C15—C14	110.1 (2)
C4—C8—H8B	109.5	C16—C15—H15A	109.6
H8A—C8—H8B	109.5	C14—C15—H15A	109.6
C4—C8—H8C	109.5	C16—C15—H15B	109.6
H8A—C8—H8C	109.5	C14—C15—H15B	109.6
H8B—C8—H8C	109.5	H15A—C15—H15B	108.2
C11—C9—C12	107.79 (16)	N1—C16—C15	109.08 (18)
C11—C9—C10	107.87 (19)	N1—C16—H16A	109.9
C12—C9—C10	109.61 (18)	C15—C16—H16A	109.9
C11—C9—C6	111.95 (16)	N1—C16—H16B	109.9
C12—C9—C6	110.53 (17)	C15—C16—H16B	109.9
C10—C9—C6	109.03 (15)	H16A—C16—H16B	108.3
C9—C10—H10A	109.5		
			
O1—C1—C2—C3	178.97 (16)	C16—N1—C7—C2	−166.73 (16)
C6—C1—C2—C3	0.0 (3)	C3—C2—C7—N1	−142.23 (18)
O1—C1—C2—C7	−5.4 (3)	C1—C2—C7—N1	42.2 (2)
C6—C1—C2—C7	175.57 (16)	C5—C6—C9—C11	−3.1 (2)
C1—C2—C3—C4	0.6 (3)	C1—C6—C9—C11	177.96 (17)
C7—C2—C3—C4	−175.06 (16)	C5—C6—C9—C12	−123.27 (19)
C2—C3—C4—C5	−0.1 (2)	C1—C6—C9—C12	57.8 (2)
C2—C3—C4—C8	179.57 (19)	C5—C6—C9—C10	116.2 (2)
C3—C4—C5—C6	−1.0 (3)	C1—C6—C9—C10	−62.8 (2)
C8—C4—C5—C6	179.35 (17)	C14—O2—C13—N1	−63.1 (2)
C4—C5—C6—C1	1.5 (3)	C16—N1—C13—O2	62.4 (2)
C4—C5—C6—C9	−177.50 (16)	C7—N1—C13—O2	−173.52 (16)
O1—C1—C6—C5	−179.96 (17)	C13—O2—C14—C15	59.0 (3)
C2—C1—C6—C5	−0.9 (2)	O2—C14—C15—C16	−54.6 (3)
O1—C1—C6—C9	−1.0 (2)	C13—N1—C16—C15	−56.7 (2)
C2—C1—C6—C9	178.05 (16)	C7—N1—C16—C15	179.72 (18)
C13—N1—C7—C2	70.2 (2)	C14—C15—C16—N1	53.5 (2)

### Hydrogen-bond geometry (Å, °)

**Table:**

*D*—H···*A*	*D*—H	H···*A*	*D*···*A*	*D*—H···*A*
O1—H1···N1	0.85 (1)	1.90 (2)	2.665 (2)	149 (3)
